# Analyzing Fault Reactivation Behavior Using InSAR, Stress Inversion, and Field Observations During the 2025 Sındırgı Earthquake Sequence, Simav Fault Zone, Western Türkiye

**DOI:** 10.3390/s26030760

**Published:** 2026-01-23

**Authors:** Şenol Hakan Kutoğlu, Mustafa Softa, Elif Akgün, Murat Nas, Savaş Topal

**Affiliations:** 1 Department of Geomatics Engineering, Faculty of Engineering, Zonguldak Bülent Ecevit University, 67100 Zonguldak, Türkiye; shakan.kutoglu@beun.edu.tr; 2 Department of Geological Engineering, Dokuz Eylül University, 35390 İzmir, Türkiye; 3 Department of Geological Engineering, Fırat University, 23200 Elazığ, Türkiye; efiratligil@firat.edu.tr; 4 Department of Civil Engineering, Karadeniz Technical University, 61080 Trabzon, Türkiye; muratnas@ktu.edu.tr; 5 Department of Geological Engineering, Pamukkale University, 20160 Denizli, Türkiye; savastopal@pau.edu.tr

**Keywords:** Sındırgı earthquake sequence, stress inversion, InSAR, simav fault zone

## Abstract

The Sındırgı earthquake sequence, with moment magnitudes of 6.1 on 10 August and 27 October 2025, respectively, occurred within the Simav Fault Zone in western Türkiye, rupturing nearby but structurally distinct fault segments. In this study, we combine Sentinel-1 InSAR time-series measurements with seismological data, geomorphic observations, and post-event field surveys to examine how deformation evolved between and after these events. InSAR results indicate coseismic line-of-sight displacements of 6–7 cm, followed by post-seismic deformation that persisted for months at 8–10 mm/yr. This behavior signifies that deformation continued well beyond the initial rupture. The estimated displacement does not align with a single fault plane. Instead, it corresponds to a network of early-mapped and previously unrecognized fault segments. Seismicity patterns and stress tensor inversions show that activity migrated spatially after 10 August and that the faulting mechanism altered before the second earthquake. When synthesized, observations indicate stress transfer within a modular, segmented fault system, thought to have been influenced by regional structural complexity. Field investigations after the October earthquake reported new surface cracks and fault traces, providing evidence of shallow deformation. The collected results indicate that post-seismic stress redistribution played a leading role in modulating the 2025 Sındırgı earthquake sequence.

## 1. Introduction

In tectonically complex regions characterized by diffuse faulting, seismic hazard assessment increasingly relies on multidisciplinary approaches that integrate geodetic, seismological, and geological observations. Such integration is particularly important where non-tectonic processes (e.g., geothermal fluid circulation or magmatic intrusions) may contribute to stress accumulation and release in the crust. While earthquakes primarily reflect tectonic fault slip, seismicity may also be influenced by fluid-driven or magmatic processes, all of which operate within a common framework of stress loading and release thereof [1,2].

Western Anatolia is one of the most seismically active regions of the eastern Mediterranean, shaped by the westward extrusion of the Anatolian microplate and ongoing Aegean extension [3,4]. Deformation in this region is accommodated by a complex network of normal and strike-slip faults, producing recurrent earthquake sequences that reflect both localized fault interactions and broader lithospheric processes. Recent large earthquakes, including the 2020 Samos event (Mw 6.9) and the 2023 Kahramanmaraş earthquake doublet (Mw 7.8 and Mw 7.6), have further demonstrated that Anatolian seismicity cannot be adequately described by simple fault geometries or isolated rupture models [5,6,7,8].

Within this context, the 2025 Sındırgı earthquake sequence provides a compelling case study of complex fault behavior in western Türkiye. The sequence was initiated by the 10 August 2025 Mw 6.1 earthquake, whereupon continued seismic activity culminating in a significant event on 27 October 2025. Although moderate in magnitude, the spatial distribution of the mainshock and aftershocks was notable, extending beyond the mapped traces of the Sındırgı segment of the Simav Fault Zone. This pattern raises questions regarding the role of secondary or unmapped faults and the possible contribution thereto of non-tectonic factors to the observed seismicity.

The Simav–Sındırgı region hosts long-recognized geothermal systems, and previous studies have documented elevated subsurface temperatures and active fluid circulation [9,10]. These conditions suggest that variations in pore-fluid pressure may locally influence fault strength and rupture behavior. Additionally, geothermal and magmatic heat input has been proposed as a mechanism for crustal weakening in western Anatolia, potentially facilitating repeated fault reactivation [11,12]. While the extent of such influences remains uncertain, their presence adds a distinct dimension to the interpretation of seismic sequences in the region.

This study investigates the 2025 Sındırgı earthquake cycle by integrating geodetic, seismological, geological, and geomorphic datasets to address four primary questions: (i) What are the characteristics of the coseismic rupture and slip distribution along the Sındırgı segment of the Simav Fault Zone? (ii) How do temporal patterns of earthquake migration and principal stress orientations reflect the active deformation regime? (iii) Why are aftershocks concentrated on the southern horst block of the Simav Mountains? (iv) Could this clustering indicate the presence of an unmapped or subsidiary fault within the broader Simav Fault Zone?

By integrating InSAR time-series analysis, stress inversion of focal mechanisms, morphometric characterization, and field mapping, this study aims to shed fresh light on resolving the rupture geometry, stress migration, and fault distribution of the 2025 Sındırgı earthquake sequence, thereby elucidating its driving processes and contributing to the broader understanding of mixed-mode diffuse deformation in the Aegean–Anatolian extensional province.

## 2. Seismotectonic Settings

**Figure 1:**
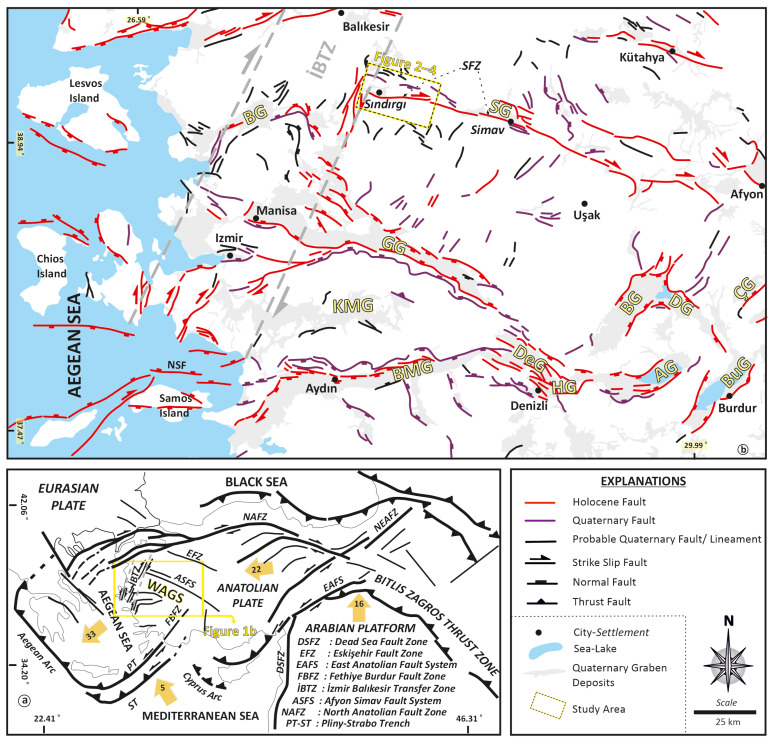
(**a**) Tectonic framework of Anatolia highlighting the Western Anatolian Graben System (WAGS) [24,25,26]. Yellow arrows denote present-day plate-motion directions, and numbers indicate relative plate velocities (mm/y) [7]. (**b**) Simplified map of western Anatolia showing the principal grabens and their bounding active faults [27,28]; offshore faults in the Aegean Sea after Ocakoğlu et al. [29], and faults bounding Samos Island after Lykousis et al. [30], Chamot-Rooke and DOTMED Working Group [31], and Chatzipetros et al. [32]. Abbreviations: BMG, Büyük Menderes Graben; GG, Gediz Graben; KMG, Küçük Menderes Graben; SG, Simav Graben; BG, Bergama Graben; BuG, Burdur Graben; AG, Acıgöl Graben; DeG, Denizli Graben; HG, Honaz Graben; BakG, Baklan Graben; DG, Dinar Graben; CG, Çölovası Graben; NSF, North Samos Fault; SFZ, Simav Fault Zone.

The Western Anatolian Graben System (WAGS) constitutes one of the most active extensional regions in the eastern Mediterranean. Since the Early Miocene, the region has experienced persistent NW–SE–oriented extension driven by Africa–Eurasia convergence and the westward tectonic escape of the Anatolian plate, with extension rates locally reaching approx. 20 mm/yr [3,4,13,14,15]. This deformation is commonly attributed to a combination of back-arc extension associated with the Hellenic subduction system, post-orogenic collapse, and lateral extrusion of Anatolia [3,16,17,18,19]. Structurally, WAGS is expressed as a series of large, fault-bounded grabens, including the Simav, Gediz, Büyük Menderes, and Küçük Menderes grabens, which together define the regional extensional architecture [20,21,22,23] (Figure 1).

Within this framework, the Simav Fault Zone (SFZ) (Figure 1) forms a key structural element of the western Afyon–Simav Fault System (ASFS), a 400 km-long (approx.) deformation belt marking the northern boundary of WAGS. The SFZ extends for approximately 214 km and is characterized by predominantly right-lateral strike-slip motion accompanied by a normal component, reflecting transtensional deformation. Based on the active fault database of Türkiye, the fault zone is subdivided into seven main segments—Sındırgı, Çaysimav, Şaphane, Abide, Banaz, Elvanpaşa, and Sinanpaşa—each exhibiting variable kinematic behavior along strike [27,28].

Kinematic constraints from field measurements, focal mechanism solutions, and earthquake relocations indicate that deformation along the Simav Fault Zone (SFZ) is governed by a regional NE–SW to NNE–SSW extensional stress regime [33,34,35,36]. Although paleoseismological and seismic studies document repeated surface-rupturing earthquakes and sustained fault activity, secondary and subsidiary structures remain poorly constrained, particularly in terms of surface expression and geodetic behavior [37,38,39]. Historical and instrumental seismicity is concentrated along the Sındırgı and Çaysimav segments, underscoring these areas as key loci of strain accumulation [40,41,42,43,44,45,46,47]. These limitations motivate the application of InSAR-based approaches; here, LiCSAR time-series products are used to systematically detect and quantify surface deformation, providing independent constraints on active and distributed faulting along the SFZ.

These recurrent events signify the persistent seismic hazard driven by the ASFS and its multisubsidiary faults. The combined structural, paleoseismological, and seismological evidence indicates that the Simav Fault Zone remains an active locus of deformation accommodating both strike-slip and extensional motions, reflecting the ongoing kinematic evolution of the Western Anatolian extensional province.

In addition to active faulting, western Anatolia exhibits widespread geothermal manifestations that are commonly associated with extensional tectonics. The Balıkesir–Sındırgı region, including the area affected by the 2025 Sındırgı earthquake sequence, hosts several geothermal occurrences that are spatially related to major fault zones within the Simav Graben and adjacent transfer structures [9,10]. Elevated heat flow and the presence of thermal springs suggest that fault-controlled permeability facilitates hydrothermal circulation in this region. While the causal relationships remain uncertain, these observations allow the hypothesis that geothermal manifestations may be linked to ongoing seismotectonic processes in western Türkiye, with potential interactions between fault activity and fluid circulation in the extensional regime.

## 3. Methodology

### 3.1. Integrated InSAR Methodology for Interseismic and Coseismic Deformation Analysis

Surface deformation related to the previously undocumented Sındırgı earthquakes along the Simav Fault Zone (SFZ) in Western Anatolia was investigated using Synthetic Aperture Radar (SAR) data acquired by the C-band Sentinel-1 satellite mission operated by the European Space Agency (ESA). Sentinel-1 enables monitoring of ground deformation at the millimeter scale regardless of atmospheric conditions or illumination geometry due to its side-looking radar imaging capability [48,49,50]. Both ascending (northwest–looking) and descending (southwest–looking) orbits were used to improve geometric constraint and sensitivity to surface motion. Interferometric pairs spanning pre-seismic and post-seismic periods were selected to retrieve long-term interseismic trends and coseismic displacement fields associated with the mainshock events.

As shown in Table 1, Sentinel-1 acquired both ascending- and descending-orbit data over the Sındırgı region during epochs that included the 10 August Mw 6.1 earthquake. The corresponding ascending and descending LOS interferograms were generated automatically by the LiCSAR system and made available through the COMET LiCSAR portal (https://comet.nerc.ac.uk/comet-lics-portal/, accessed on 28 November 2025). For the second earthquake sequence, Sentinel-1 imaged the study area in both orbit directions on 10 October 2025 and 28 October 2025, covering the coseismic period of the 27 October 2025 event. The interferograms derived from these datasets were downloaded directly from the Comet LiCSAR web interface and are summarized in Table 1.

Line-of-sight (LOS) interferograms were generated automatically using the LiCSAR frame-based processing system following Lazecký [51] and obtained from the COMET LiCSAR portal. Sentinel-1 SLC bursts intersecting the study frame were mosaicked into epoch-wise images using restituted or precise orbit data, and multilooked products (4 looks azimuth, 20 looks range; approx. 56 × 46 m resolution) were produced. Digital elevation model (DEM) assisted coregistration was performed using 1 arc-second Shuttle Radar Topography Mission (SRTM) data, refined by intensity cross-correlation and spectral diversity to correct TOPS azimuth misregistration. Differential interferograms were generated by removing the simulated topographic phase, while atmospheric effects were not corrected in the default processing. Phase unwrapping was applied after adaptive spatial filtering and coherence-based masking using snaphu (v2).

Interseismic surface deformation time series were obtained from the LiCSAR processing system, which automatically generates interferograms from Sentinel-1 acquisitions, applies precise orbit refinement, performs atmospheric mitigation, and produces geocoded products. Time-series inversion and velocity estimation were carried out using the LiCSBAS v2.0 software package, whereby phase unwrapping, temporal filtering, and noise reduction were integrated to derive displacement histories and average deformation rates [51,52,53,54]. This analysis enabled the identification of long-term strain accumulation and interseismic deformation patterns along the Simav Fault Zone.

Coseismic deformation was analyzed using Differential Interferometric SAR (DInSAR), which quantifies surface displacement by differencing the radar phase of two SAR images acquired before and after the earthquakes [55,56]. The interferometric phase contains signals from topography, orbital errors, atmospheric delays, deformation, and noise. The topographic contribution was removed using the SRTM (DEM), while orbital errors were corrected using precise orbit information. Atmospheric contributions were reduced using filtering techniques, leaving the residual phase dominated by surface displacement.

The Line-of-Sight (LOS) surface displacement (d) was calculated from the interferometric phase difference (Δφ) using

(1) $$d=\left(\frac{\lambda }{2}\right)\left(\frac{\Delta \phi }{2\pi }\right)$$

where λ is the radar wavelength (5.6 cm for Sentinel-1), each full 2π radian phase cycle corresponds to approximately half the radar wavelength in LOS displacement. This formulation allows quantitative mapping of coseismic surface deformation associated with earthquake faulting processes [57,58,59].

To reconstruct the three–dimensional deformation field, LOS displacements from ascending and descending orbits were combined using the following geometric decomposition:

(2) $$\left[\begin{array}{c} d_{LOS}^{asc}\\ d_{LOS}^{dsc} \end{array}\right]=\left[\begin{array}{c} sin\theta^{asc}cos\alpha^{asc}-sin\theta^{asc}cos\alpha^{asc}\;-cos\theta^{asc}\\ sin\theta^{asc}cos\alpha^{asc}-sin\theta^{asc}cos\alpha^{asc}\;-cos\theta^{asc} \end{array}\right]\left[\begin{array}{c} d_{E}\\ d_{N}\\ d_{U} \end{array}\right]$$

where θ denotes the local radar incidence angle, defined as the angle between the incident radar wave and the surface normal at the target location, and α represents the azimuth angle of the satellite heading (flight-direction) vector, measured clockwise from North in the local horizontal plane, in accordance with the standard InSAR geometry described in Morishita [54]. The displacement components $d_{E}$, $d_{N}$, and $d_{U}$ correspond to east–west, north–south, and vertical motions, respectively. Owing to the limited north–south sensitivity inherent to the Sentinel-1 orbital geometry, the interpretation primarily focuses on the east–west and vertical displacement components.

Quality control was carried out by removing areas with low interferometric coherence, where reliable deformation measurements cannot be obtained. Displacement uncertainty was evaluated using reference areas assumed to be stable and unaffected by surface motion. In addition, deformation fields derived from ascending and descending satellite orbits were compared, and their agreement was used as an independent check on the reliability of the results.

### 3.2. Stress Inversion

The present study investigates the regional stress field along the Sındırgı segment of the Simav Fault Zone (SFZ) using present-day stress inversion of earthquake focal mechanism solutions (FMS). This approach allows reconstruction of the principal stress axes ($\sigma_{1}$, $\sigma_{2}$, $\sigma_{3}$) and estimation of the stress ratio, $R=\frac{\sigma_{2}-\sigma_{3}}{\sigma_{1}-\sigma_{3}}$, enabling quantitative interpretation of fault kinematics and stress distribution within the study area.

Focal mechanism solutions were retrieved from national seismic catalogs compiled by the Disaster and Emergency Management Authority of Türkiye [60] and include well-constrained solutions distributed along the Simav Fault Zone. Only events with reliable polarity data, sufficient station coverage, and low uncertainty in nodal plane determination were used. The method assumes that coseismic slip occurs parallel to the direction of maximum resolved shear stress acting on each fault plane [61,62], and that the stress field is approximately homogeneous within defined tectonic domains.

Stress tensor inversion was performed using the Rotational Optimization Method [63] in Win-Tensor v6.0.1. software. This algorithm systematically rotates the stress tensor to minimize the angular misfit between observed slip vectors and theoretical shear directions predicted by the model. The inversion procedure provides optimal orientations of the three principal stress axes, along with the stress ratio (R), which reflects the relative magnitudes of the principal stresses and indicates the tectonic regime. Unstable or poorly constrained solutions were excluded based on convergence behavior and statistical reliability. An initial stress tensor was first estimated using the Improved Right Dihedron method and subsequently refined using the Rotational Optimization algorithm, which applies a controlled four-dimensional grid search involving successive rotations of the principal stress axes (σ_1_, σ_2_, σ_3_) and systematic testing of the stress ratio R. The inversion employed initial rotation ranges of ±45° and R values between 0 and 1, progressively narrowed to ±5° and ±0.1, respectively, until convergence was achieved and the misfit function could no longer be reduced [63].

Because focal mechanisms yield two possible nodal planes, fault plane ambiguity was resolved using additional geological and geophysical constraints, including mapped fault traces, regional seismotectonic frameworks, earthquake alignments, and known fault kinematics derived from field studies and satellite observations. When independent constraints were insufficient, both nodal planes were tested, and the solution producing the lowest misfit was selected [64].

Focal mechanisms were classified according to the Frohlich [65] diagram to distinguish extensional, strike-slip, and compressional regimes. Stress regimes were inferred from the relative ordering of principal stress axes and stress ratio values. Solutions with mean misfit angles less than 20° and high internal consistency were considered reliable and used to delineate variations in stress orientation and regime along the Sındırgı segment.

To examine spatial variations in the stress field, the dataset was subdivided into segments according to fault geometry and earthquake clustering. Inversion results were analyzed to identify possible stress rotations, kinematic transitions, and mechanical segmentation along the fault system. Finally, stress results were integrated with geomorphological observations and InSAR-derived deformation patterns obtained from remote sensing to establish a comprehensive interpretation of crustal deformation along the Simav Fault Zone.

### 3.3. Channel Steepness Analysis and Structural Field Mapping

Active deformation along the Sındırgı segment of the Simav Fault Zone was evaluated using normalized channel steepness (ksn) to identify spatial variations in fault-related uplift, supported by targeted structural field observations. This approach emphasizes tectonic signals expressed in topography rather than detailed geomorphic classification.

Normalized channel steepness indices were calculated from a 12.5 m resolution ALOS PALSAR digital elevation model using standard hydrological processing and MATLAB^®^ R2023b-based TopoToolbox Version 3 routines [66,67,68,69,70]. Elevated ksn values were evaluated in relation to mapped fault traces and regional structural trends. Anomalous ksn zones lacking correspondence with known faults were subsequently targeted for field investigations, where fault scarps, displaced geomorphic markers, linear fronts, and fracture zones were documented. Structural measurements and field mapping confirm that these ksn anomalies correspond to zones of active or recently reactivated faulting. Field mapping led to the identification of several previously unmapped fault traces, which were incorporated into an updated structural framework for the Sındırgı region, demonstrating that the combined *k*_sn_ analysis and field verification effectively capture distributed deformation along the Simav Fault Zone.

## 4. Result

### 4.1. LiCSAR-Derived Surface and Coseismic Deformation Patterns

**Figure 2:**
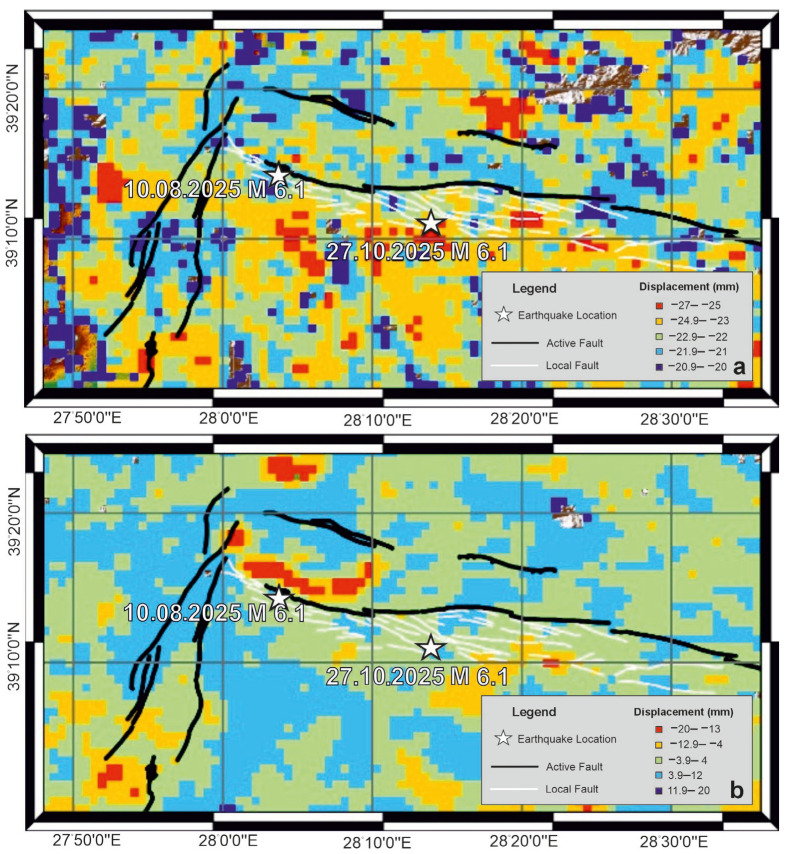
East–West (**a**) and vertical (**b**) surface velocities between 2015 and 2019 from LiCSAR/LiCSBAS solution. Active fault traces from Emre et al. [28] are shown by black solid lines, while white solid lines indicate faults newly mapped in this study.

Long-term interseismic deformation patterns of the Simav Fault Zone (SFZ) were evaluated using LiCSAR/LiCSBAS velocity products previously published by Weiss et al. [71], which provide 1 km × 1 km resolution east–west and vertical velocity fields for the whole of Anatolia for the period 2015–2019. Based on these data, east–west and vertical velocity maps for the Sındırgı region are presented in Figure 2a,b, respectively.

As shown in Figure 2a, east–west interseismic surface displacements across the fault zone are limited to approximately 1–2 mm/yr. Although the signal is relatively weak, the southern block exhibits a westward motion of about 2 mm/yr relative to the northern block. This spatial pattern indicates the presence of a right-lateral (dextral) strike-slip component along the fault. However, the magnitude of this horizontal displacement is small, suggesting that lateral motion is not the dominant mode of deformation during the interseismic period. In contrast, vertical velocity patterns (Figure 2b) show a pronounced signal, especially around the segment hosting the first mainshock. Vertical motion reaches approximately −2 cm/yr on the northern side of the fault relative to the southern side, indicating downward motion of the hanging wall and a clear normal faulting component thereon. This vertical displacement pattern weakens eastward toward the epicentral area of the second earthquake and becomes indistinct thence. Overall, interseismic deformation results indicate that the regional tectonic regime is dominated by normal faulting, with a minor but detectable right-lateral strike-slip component.

**Figure 3:**
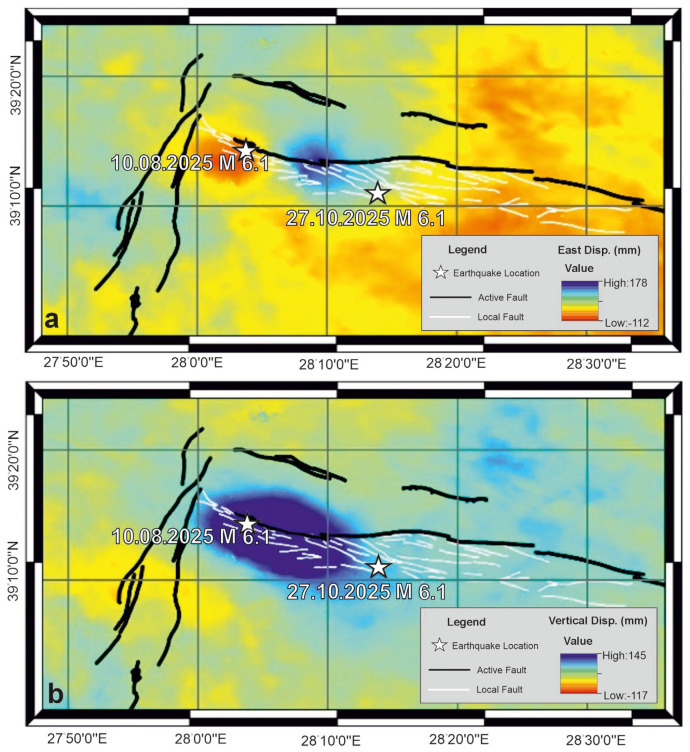
East–West (**a**) and Vertical (**b**) coseismic displacement field associated with the 10 August Mw 6.1 earthquake. Active fault traces from Emre et al. [28] are shown by black solid lines, while white solid lines indicate faults newly mapped in this study.

Coseismic deformation associated with the 10 August Mw 6.1 earthquake was analyzed by decomposing LiCSAR-derived Line-of-Sight (LOS) displacements into east–west and vertical components using Equation (2). The resulting coseismic displacement maps are shown in Figure 3a,b. Figure 3a reveals an opposite horizontal motion across the main fault trace: the eastern block moved eastward by up to 11 cm, while the western block shifted westward by approximately 10 cm. The total horizontal offset thus reaches approx. 21 cm, clearly indicating a dominant right-lateral strike-slip mechanism. Vertical coseismic displacement associated with this event is presented in Figure 3b. A strong vertical signal is observed, with maximum displacement reaching 14 cm. The combined horizontal and vertical displacement patterns demonstrate that the 10 August quake occurred under oblique-slip kinematics, characterized by both dextral strike-slip and normal faulting components.

**Figure 4:**
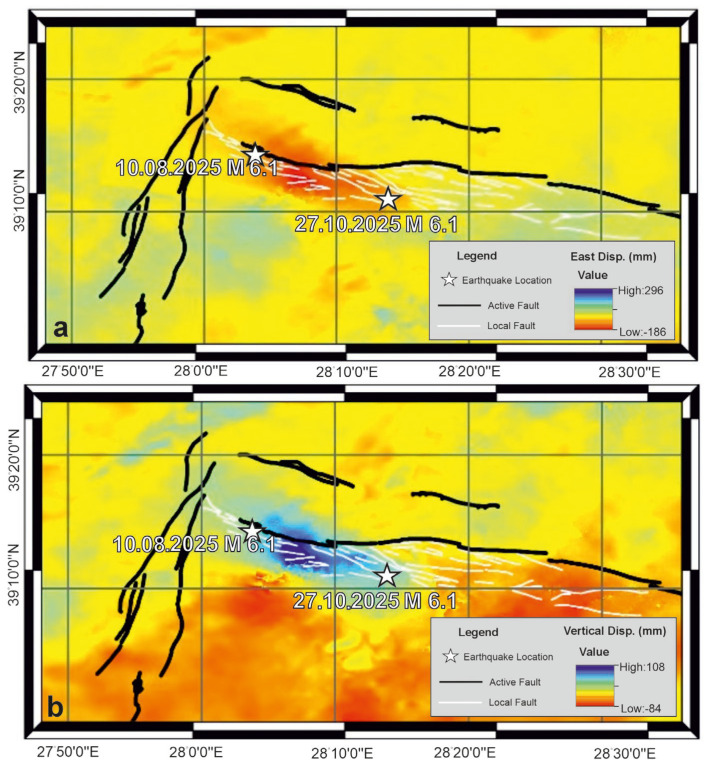
East–West (**a**) and vertical (**b**) coseismic displacement field associated with the 27 October Mw 6.1 earthquake. Active fault traces from Emre et al. [28] are shown by black solid lines, while white solid lines indicate faults newly mapped in this study.

Coseismic deformation from the 27 October Mw 6.1 earthquake exhibits distinct kinematic behavior. East–west displacement patterns (Figure 4a) indicate nearly pure westward motion, with a maximum displacement of approximately 11 cm. Unlike the first event, a slight eastward displacement is observed, suggesting a predominantly unilateral horizontal motion. Vertical displacement distribution for the 27 October event is shown in Figure 4b. Although weaker than in the first event, vertical motion is still evident, with a maximum displacement of 7.4 cm. These results suggest that, while the second earthquake was primarily strike-slip, it also included a modest normal-fault component.

In summary, the interseismic velocity field reveals a tectonic regime dominated by normal faulting with a weak strike-slip component. In contrast, coseismic deformation results indicate that both earthquakes occurred due to oblique-slip faulting, combining right-lateral strike-slip and normal components, with notable variation in kinematic behavior between the two events.

### 4.2. Present-Day Stress Fields from Seismic Data

The spatiotemporal distribution of earthquake sequences in western Anatolia reveals a heterogeneous deformation pattern, expressed by distinct seismic phases and systematic variations in fault kinematics. Within this framework, the Sındırgı and Çaysimav segments of the Simav Fault Zone (SFZ), together with the Gelenbe Fault Zone (GFZ), display a series of temporally discrete yet mechanically interconnected seismic phases, indicative of progressive stress redistribution between adjacent fault segments.

**Figure 5:**
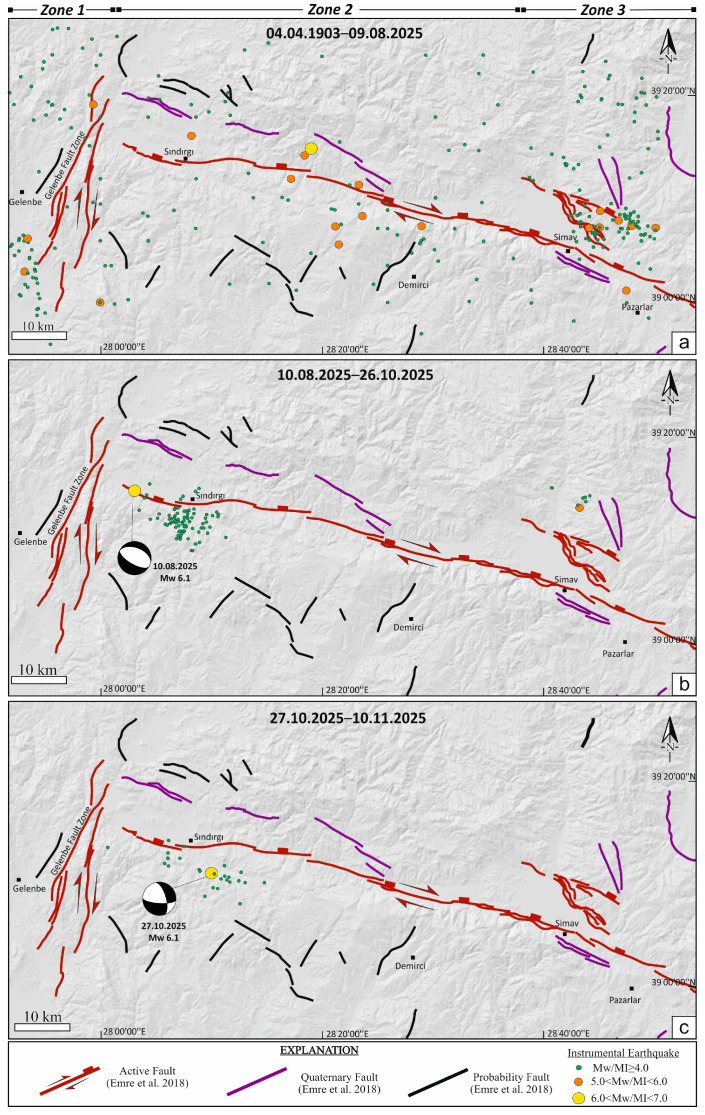
Spatiotemporal evolution of seismicity along the SFZ and GFZ within Western Anatolia from 1903 to 2025. Panels (**a**–**c**) show long-term seismicity, the 10 August 2025, Mw 6.1 event, and the 27 October 2025, Mw 6.1 event, respectively. Faults are from Emre et al. [28], and earthquakes are color-coded by magnitude; focal mechanisms (represented as beach ball diagrams, where white areas indicate tension and black areas indicate compression) correspond to the two Mw 6.1 events obtained from [60]. Earthquake epicenters coincide spatially with areas of previously unmapped structures, potentially related to the Sındırgı segment of the Simav Fault Zone (SFZ) within the Simav Mountains horst.

The first phase, initiated by the 19 May 2011, MI 5.7 Simav earthquake [60], produced MI ≥ 4.0 aftershocks distributed across both the SFZ and GFZ. A subsequent MI 5.4 event on 3 May 2012 [60], further intensified aftershock clustering, indicating the progressive redistribution of stress along these fault systems. A second seismic phase became evident with the revival of activity on the GFZ in late 2019, culminating in the Mw 5.4 Akhisar main–shock on 22 January 2020, and followed by the Mw 5.2 Kırkağaç (18 February 2020) and Mw 5.5 Saruhanlı (26 June 2020) earthquakes [60]. These events illustrate a stepwise east–west migration of moderate-magnitude earthquakes, consistent with episodic stress transfer between structurally linked fault segments (Figure 5a).

A third and more pronounced seismic phase unfolded during 2025, beginning with MI ≥ 4.0 activity along the Çaysimav segment and peaking with the Mw 6.1 main-shock on 10 August 2025 [60], at the northwestern termination of the Sındırgı segment within the SFZ. The normal-faulting focal mechanism of this event confirms the dominance of extensional deformation. Aftershock propagation toward the east, contrasted with its inhibition toward the west by the NE–SW–oriented GFZ, demonstrates the GFZ’s role as a mechanical barrier (Figure 5b). The largest aftershock of this sequence (Mw 5.4 on 28 September 2025, near Simav) and a second Mw 6.1 main-shock on 27 October 2025 [60] further support the notion of eastward-directed stress transfer and a multi-stage seismic event within the SFZ and GFZ–fault system (Figure 5c).

**Figure 6:**
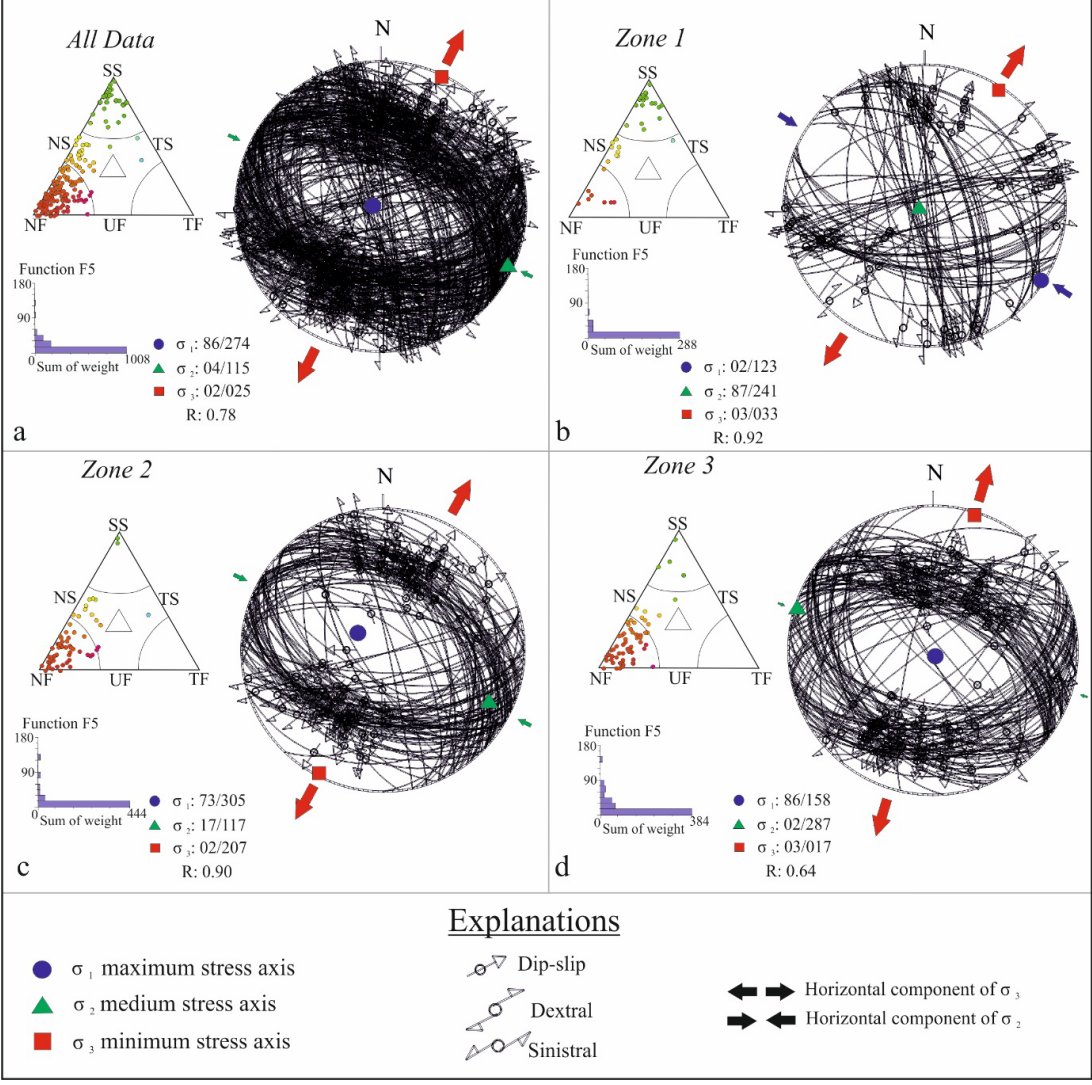
Results of stress tensor inversion calculated using the Win-Tensor software v.6.0.1 [63,64]. Panel (**a**) shows the regional inversion based on the complete dataset, whereas panels (**b**–**d**) present solutions for Zones 1, 2, and 3, respectively. Corresponding triangular diagrams from Frohlich [65] are included to illustrate faulting mechanisms and inferred stress regimes. Colors of the data points within the Frohlich ternary diagram indicate the dominant faulting style inferred from focal mechanisms: green—strike-slip faulting; yellow to orange—oblique strike-slip with a normal faulting component; and red to pink—normal faulting.

To characterize the contemporary stress field associated with these events, focal mechanism solutions from the 2010–2025 interval were inverted and classified into three tectonic domains: Zone 1 (GFZ), Zone 2 (Sındırgı segment within the SFZ), and Zone 3 (Çaysimav segment within the SFZ). The inversion results reveal systematic but domain-specific stress patterns (Table 2). Stress inversion of the complete dataset reveals a regionally dominant extensional stress regime, with the principal extension trending approximately NE–SW. The best-fit stress tensor yields a stress regime index of R = 0.78 ± 0.21, consistent with a transtensional tectonic regime. The minimum horizontal principal stress (SHmin) is oriented at 25° ± 12.3°, further substantiating an ENE–WSW extension setting. The principal stress axes are oriented as follows: σ_1_ (maximum compressive stress) = 86°/274°, σ_2_ (intermediate stress) = 04°/115°, and σ_3_ (minimum compressive stress/extensional axis) = 02°/025°. These values suggest a coherent extensional stress regime across the SFZ and GFZ that well coincides with observed seismotectonic trends and fault geometries (Figure 6a). Zone 1 is dominated by a transtensional stress tensor, marked by NW–SE compression (SHmax: 122° ± 10.8°) paired with NE–SW extension (SHmin: 32° ± 10.8°), reflecting the strike-slip to oblique-slip kinematics of the GFZ (Figure 6b). Zone 2 exhibits a predominantly extensional stress regime compatible with a transtensional tectonic regime index, again characterized by NW–SE compression (SHmax: 117° ± 11.2°) coupled with NE–SW extension (SHmin: 27° ± 11.2°), indicative of normal faulting with minor dextral strike-slip components along the Sındırgı segment within the SFZ (Figure 6c). Zone 3 displays a purely extensional stress state, consistent with the normal-fault–dominated kinematics (SHmin: 17° ± 16.5°) along the Çaysimav segment of the SFZ (Figure 6d). When integrated, these stress orientations confirm that the region is undergoing a transtensional–to–extensional deformation continuum, with seismicity evolving in multi-phase cycles driven by the redistribution of strain across fault zones within the Western Anatolian extensional deformation region.

### 4.3. Channel Steepness and Structural Constraints on Active Deformation

**Figure 7:**
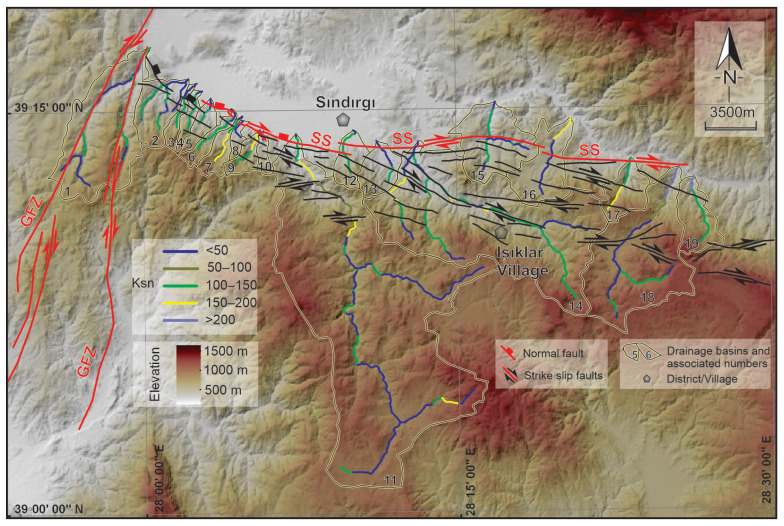
Distribution of morphometric indices measured along the principal river channels within drainage basins developed on the southern branches of the Sındırgı Segment overlaid with a shaded relief map. ksn: normalized channel steepness index; GFZ: Gelenbe Fault Zone; SS: Sındırgı Segment. Active fault traces from Emre et al. [28] are shown by red solid lines, while black solid lines indicate faults newly mapped in this study.

To evaluate spatial variations in active deformation along the Sındırgı segment of the Simav Fault Zone (SFZ), we analyzed normalized channel steepness (ksn) patterns and integrated these results with targeted structural field observations. This approach provides an independent constraint on fault-related deformation associated with the 2025 earthquake sequence (Figure 7).

Normalized channel steepness values exhibit strong spatial variability along the fault zone. High Ksn values (>150) are concentrated along the southern footwall and in channels draining toward the main fault trace, whereas lower values (<100) dominate more distal parts of the drainage network. Sharp ksn gradients coincide with channel-slope breaks aligned with mapped fault traces, indicating that fluvial profiles are strongly influenced by fault-controlled deformation.

**Figure 8:**
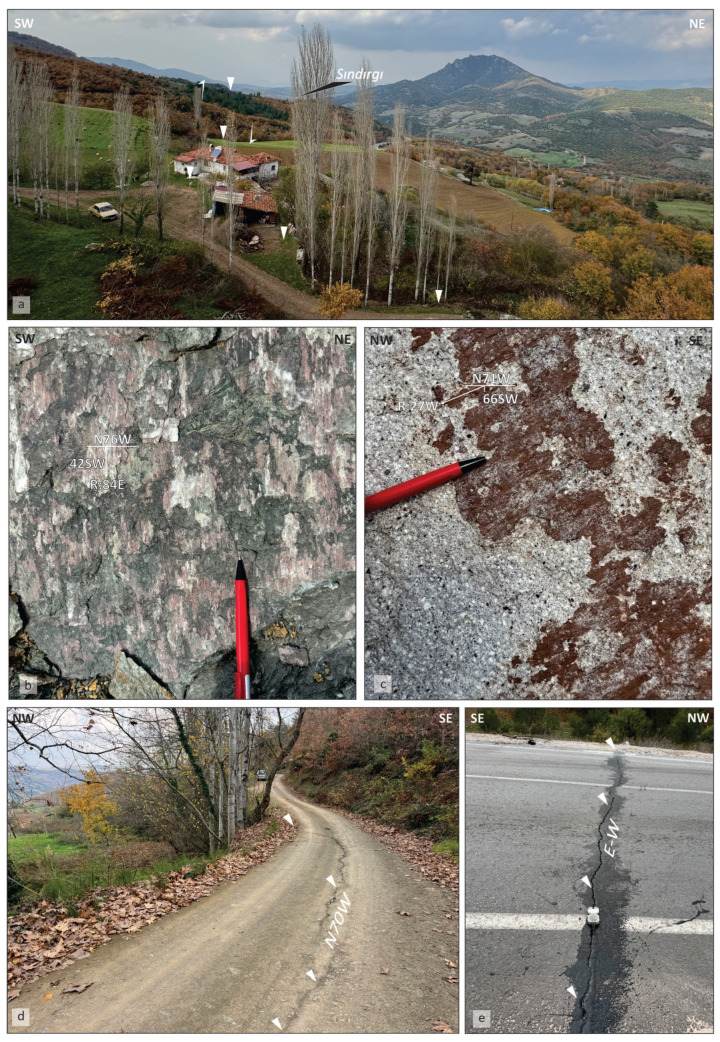
Morphological expression of the newly mapped Sındırgı segment between Karataş and Sındırgı. (**a**) General view of fault-related geomorphic features along the fault zone (UTM Zone 35S: 609523E, 4337480N). (**b**) Close-up view of an approximately NW–SE–striking normal fault plane with dip-slip slickenlines, marked by calcite fibers (UTM Zone 35S: 596931E, 4340762N). (**c**) Close-up view of an approximately NW–SE–striking fault plane with an oblique to strike-slip sense of motion, indicated by mineralized slickenlines within volcanic rocks (UTM Zone 35S: 605368E, 4341121N). (**d**) NW–SE–oriented surface crack outcrops developed during the Sındırgı earthquake sequence in Karataş town (UTM Zone 35S: 609551E, 4337412N). (**e**) E–W–oriented surface cracks observed on the asphalt road (UTM Zone 35S: 609150E, 4338037N). White arrows highlight the observed morphological lineaments and their sense, as identified in the field.

Elevated Ksn values were used as first–order indicators and interpreted only where they coincide with independent structural evidence, given that k_sn_ anomalies may also reflect nontectonic effects such as lithologic variability or transient channel adjustment. Field investigations of these zones document concentrated faulting and resulted in the mapping of several previously unmapped fault traces along the western and central Sındırgı segment. These structures define a discontinuous fault network that influences drainage organization and surface deformation (Figure 8a–c). Structural measurements show systematic along-strike variations in fault kinematics, from predominantly dip-slip motion in the west to oblique–strike-slip motion toward the east. Further, surface fractures documented following the 27 October 2025 Mw 6.1 earthquake indicate shallow deformation within the fault zone. Field mapping in the Işıklar village recorded crack orientations predominantly around N70° W with local E–W deviations, consistent with the strikes of faults mapped in this study (Figure 8d,e). The spatially distributed pattern of surface cracking, together with the newly mapped fault traces, indicates deformation accommodated across multiple structures rather than localized on a single fault plane. These field observations are consistent with LiCSAR InSAR time-series results, which show distributed surface deformation and support a segmented fault geometry for the Sındırgı segment during the 2025 earthquake sequence.

## 5. Discussion

### 5.1. Interpretation of Interseismic–Coseismic Deformation Patterns

Interseismic and coseismic deformation patterns derived from Sentinel-1 InSAR observations provide new constraints on the kinematics of the Sındırgı segment of the Simav Fault Zone (SFZ).

In this area, earthquake mechanisms and long-term fault behavior have not been thoroughly examined. The LiCSAR/LiCSBAS velocity data combined with DInSAR measurements of coseismic displacement show that slip varies considerably across the area. This variation points to a complicated interaction between strike-slip and normal faulting.

Long-term deformation along the fault is mainly dominated by a normal-faulting regime, as evidenced by interseismic velocities from LiCSBAS. Dominant expansion across the zone is indicated by the northern side of the fault subsiding at rates close to 2 cm/yr in comparison to the southern side. This trend is rather consistent with the larger geodynamic picture of Western Anatolia, where NE–SW crustal stretching is notably driven by continuous Aegean back-arc extension. A secondary, right-lateral strike-slip component is evidenced by the slightly faint yet observable westward migration of the southern block (2 mm/yr) relative to the northern one. This kind of horizontal deviation is also common in diffused transtensional systems, where regional extension is dominant but shear is widely accommodated along locally pre-existing structures. The limited lateral deformation signature, however, implies that interseismic shear accumulation is either minor or highly localized.

The coseismic deformation associated with the Mw 6.1 earthquake of 10 August 2025 displays a displacement pattern that differs from that of the Mw 6.1 event of 27 October 2025. Geodetic observations indicate that the August event is characterized by a pronounced east–west–oriented bilateral displacement field, with cumulative horizontal offsets of up to 21 cm. A significant vertical component, locally exceeding 14 cm, accompanies this motion, indicating oblique slip with combined strike-slip and normal components. In contrast, the October event produced a more spatially uniform westward displacement of approximately 11 cm and a comparatively weaker vertical signal (7.4 cm), suggesting a different balance between horizontal and vertical slip components.

Although both earthquakes occurred within the broader tectonic framework of the Western Anatolian Graben System, their coseismic deformation patterns are not identical. Rather than implying a uniform rupture behavior, these observations are consistent with the hypothesis that the two events activated different fault segments, different portions of the same segmented fault system, or experienced distinct local stress conditions at the time of rupture. In this context, the 10 August event cannot be interpreted as a purely strike-slip earthquake in the same sense as the 27 October event but instead reflects a more strongly oblique rupture style.

The contrast between the predominantly normal-faulting deformation inferred from long-term geodetic observations and the strike-slip–dominated components observed during some coseismic ruptures raises questions regarding strain accumulation within the Simav Fault Zone. One further yet possible corollary is that shear strain may accumulate heterogeneously within the fault system, potentially at depth or within mechanically distinct fault segments that are not fully expressed in surface deformation. Such behavior could arise from variations in fault locking depth, along-strike segmentation, or spatial heterogeneity in frictional properties. At present, this interpretation remains qualitative, as a quantitative assessment of slip deficit and strain partitioning is beyond the scope of the available data. Additional processes may also influence coseismic rupture behavior. The Sındırgı region hosts active geothermal systems, and fluid circulation within the crust may locally modify effective stress conditions along faults. Elevated pore-fluid pressures could dramatically reduce fault strength that influences the rupture initiation or its propagation pattern. Likewise, localized magmatic or thermal perturbations, if present, may alter the stress field on short spatial or temporal scales. While such mechanisms have been documented in other extensional and volcanic settings, their role in the 2025 Sındırgı earthquake sequence remains intensely speculative. It is therefore presented here as a plausible working hypothesis rather than a demonstrated sole cause.

The coexistence of extension tectonics, geothermal indicators, and irregular coseismic behavior suggests that magmatic involvement in the Sındırgı region cannot be directly confirmed geophysically at present (e.g., via seismic tomography or magnetotelluric methods). It indicates that the earthquake sequence may have profoundly been influenced by both tectonic loading and fluid-induced transient stresses. Further developments in heat-flow measurements, seismic activity studies, and the integration of chemical records with InSAR are of great importance for supporting this hypothesis.

The pattern of deformations in and around the Sındırgı area suggests that the Simav Fault Zone behaves as a transtensional fault, in which the relative dominance of extension versus right-lateral shear may change in different regions and times. The combination of tectonic stress build-up and possible fluid-related reduction in resistance mechanisms not only emphasizes the complexities engaged in earthquake diffusion into the area, but it also emphasizes the importance of getting a convergence in multi-temporal InSAR analyses with multidisciplinary geophysics observation.

**Figure 9:**
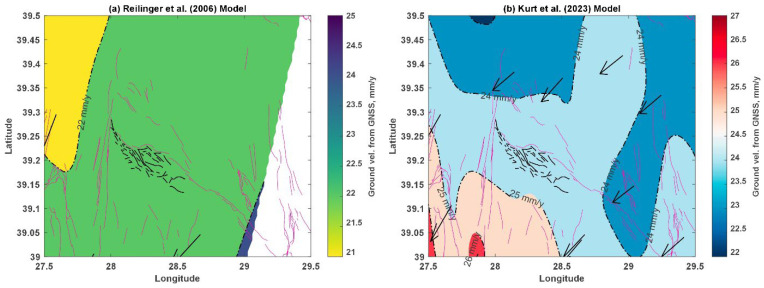
Ground velocity fields for the study area: (**a**) velocity distribution after Reilinger et al. [7], (**b**) velocity distribution after Kurt et al. [73]. Arrows indicate deformation vectors and their directions. Active fault traces from Emre et al. [28] are shown by red solid lines, while black solid lines indicate faults newly mapped in this study.

To understand the observed deformation, a reliable, long-term dataset was required. These data provide a fundamental point of reference, while GNSS measurements are particularly well-suited to this use. Nevertheless, as the number of these studies is not large enough to examine crustal movement across Türkiye’s entire geographic area, and most research [2,7,72,73] has been influenced by major contributions such as those of Reilinger et al. [7] and Kurt et al. [73]. The basic information gathered from these seminal investigations was reviewed for this investigation. Instead of being adopted directly, published velocity and strain rate statistics were reprocessed to ensure temporal consistency and achieve the required geographical coverage. Consequently, a comprehensive, up-to-date picture of regional ground motion was obtained. The Sındırgı Segment was positioned at the center of new velocity maps derived from the reprocessed dataset, enabling an evaluation of its role within a more comprehensive tectonic framework. The spatial distribution of annual ground velocities within the study area and its surrounding region is shown in Figure 9. The reprocessed version of the Reilinger et al. [7] model is given in Figure 9a. It provides a broad, unified deformation zone for the study area and its vicinity. The data is found to be lacking on the east side of the Sındırgı Segment. However, a clear distinction is evident along the Çaysimav Segment. According to Reilinger et al. [7], the study area is within the 22 mm/y. Figure 9b is based on a reprocessed version of the ground velocity dataset originally presented by Kurt et al. [73]. It covers the study area and beyond, as expected, now that it is made for the full extent of Türkiye. Kurt et al. [73] provide fairly enhanced spatial coverage of the study area. The better resolution of this data facilitates more reliable interpretations within the study region and its surroundings. It is clear that the Çaysimav Segment is separated from the Sındırgı Segment, both of which belong to distinct geologic settings with distinct behaviors. The annual relative velocity is found to be 24 mm/y, which is 2 mm/y greater than the prior. For each model tested, the deformation vector and its azimuth angle are almost the same. It shows that, despite roughly 17 years between these referenced studies, the behavioral pattern has not changed, but a 2 mm/y increase is observed.

### 5.2. Integrated Interpretation of Stress Regimes and Multi-Phase Seismic Evolution

**Figure 10:**
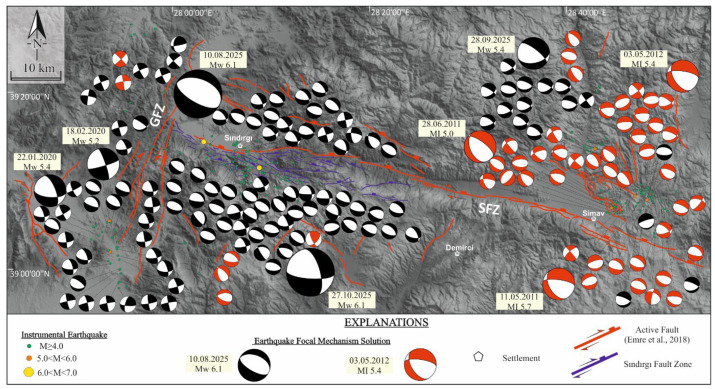
Distribution of instrumental earthquakes (Mw ≥ 4.0) recorded between 2010 and 2025 along the fault systems within the Western Anatolian region, including focal mechanism solutions, based on AFAD data [60]. Fault segments and related structures are labeled as follows: GFZ-Gelenbe Fault Zone, SFZ-Simav Fault Zone. Active fault traces from Emre et al. [28] are shown by red solid lines, while purple solid lines indicate faults newly mapped in this study.

Western Anatolia has undergone a long-lived and spatially complex tectonic evolution associated with crustal extension and uplift within the broader Western Anatolian Graben System. Earlier phases of deformation involved strike-slip and transtensional regimes that contributed to the exhumation of metamorphic massifs and the development of regional horst–graben architecture [74,75,76,77]. These structures were subsequently reactivated under a more pronounced extensional stress field related to the retreat of the Hellenic subduction zone, resulting in the present-day fault network dominated by normal and oblique-slip faulting [4,21,78,79]. Following the 2025 Sındırgı earthquake sequence, aftershock activity was concentrated mainly along the southern part of the Sındırgı segment, as documented in existing fault maps [28]. The spatial distribution of aftershocks is consistent with the presence of short, WNW–ESE–oriented fault strands that connect to or run subparallel to the main fault segment (Figure 10). One possible interpretation is that these structures represent mechanically mature fault elements that were preferentially reactivated during the sequence. However, the extent to which these faults influenced rupture propagation or segmentation cannot be resolved with the available data and therefore remains a working hypothesis. Elevated geothermal gradients and evidence for high subsurface temperatures in the Sındırgı region [11] introduce additional complexity to the interpretation of fault behavior. High temperatures may reduce crustal strength and influence fault rheology, potentially affecting how stress is accommodated during both interseismic and coseismic periods. Analogous observations from volcanic and geothermal regions, such as the Santorini system [12], suggest that thermal anomalies can locally modify stress conditions. In the Sındırgı case, such effects are considered as a possible contributing factor rather than a demonstrated control on seismicity. The concentration of Mw 6.1 earthquakes within the horst block and the partial mismatch between some focal mechanism solutions and mapped fault traces further indicate that deformation may not be confined to a single, planar fault geometry. One hypothesis is that localized thermal or fluid-related weakening could promote rupture on secondary or oblique structures that are not optimally oriented with respect to the long-term regional stress field. Alternatively, these discrepancies may reflect fault segmentation, depth-dependent rupture behavior, or limitations in surface fault mapping. At present, these interpretations remain qualitative and cannot be distinguished unambiguously.

Collectively, the observations from the 2025 Sındırgı earthquake sequence suggest that deformation in the region may result from the interaction of regional extension, fault segmentation, and local rheological heterogeneities. Rather than indicating a single dominant mechanism, the seismic behavior of the Sındırgı segment is consistent with a multi-component system in which tectonic forcing, structural complexity, and possible thermal effects collectively influence rupture style and stress release. These interpretations are presented as hypotheses that are contingent thereon and straightforwardly highlight the need for further quantitative constraints from geodesy, seismic imaging, and thermal modeling when assessing active deformation and seismic hazard in Western Anatolia. The multi-phase spatiotemporal evolution of seismicity along the Çaysimav and Sındırgı segments of the SFZ–GFZ system between 2010 and 2025 is broadly consistent with the structural framework and kinematic diversity previously described for the Simav Fault Zone. Earlier studies highlighted that the SFZ comprises several segments with mixed strike-slip and normal-faulting behavior [27,28], with the western Sındırgı segment showing dominant dip-slip motion and subordinate dextral components. Our results corroborate this segmental kinematic variability and further demonstrate that these structural differences exert a primary control on the partitioning and migration of seismicity over decadal timescales.

The three distinct seismic phases identified, 2011–2012, 2019–2020, and 2025, suggest a cyclic reactivation pattern regulated by episodic stress transfer between mechanically linked fault segments. Notably, the inhibited westward aftershock migration during the 2025 Mw 6.1 sequence emphasizes the role of the NNE–SSW–striking GFZ as a mechanical barrier, which is an ongoing behavior that has not been highlighted so far, depending on the region-specific tectonic interpretations.

The stress inversion results presented here confirm a regionally dominant NE–SW extensional regime, consistent with earlier focal mechanism analyses from the 1996 and 2011 Simav earthquakes [34,36]. However, our more extensive and up–to–date dataset refines this understanding by revealing domain-specific stress variations: the GFZ exhibits a transtensional stress tensor with NW–SE compression; the Sındırgı segment shows mixed normal faulting with a minor dextral component; and the Çaysimav segment displays purely extensional kinematics. This dominant stress variability is consistent with structural heterogeneity described by Gündoğdu et al. [33,35]. However, our results indicate a more broadly distributed NE–SW extensional regime with localized transtensional overprints near apparent geometric complexities.

Furthermore, the E–W migration of moderate-to-large earthquakes between 2011 and 2025 aligns with the presence of listric geometries and distributed normal faulting within the SFZ, as proposed for the 1996 Simav sequence [36] and the Şaphane–Gürlek substructures [37]. These structural configurations likely facilitate strain redistribution toward the eastern SFZ segments, promoting the sequential activation pattern observed during the 2025 Mw 6.1 doublet.

When synthesized, the interplay of multi-phase seismicity, domain-specific stress fields, and structural segmentation indicates that the SFZ–GFZ system operates as a transtensional fault network undergoing repeated cycles of strain accumulation and release. This integrated perspective highlights the critical need for high-resolution mapping, systematic paleoseismological investigations, and continuous geodetic monitoring of the Sındırgı segment and adjacent horst-bounding faults, which remain insufficiently characterized despite their demonstrated high seismic potential.

### 5.3. Channel Steepness Patterns and Field Evidence of Shallow Deformation near the Sındırgı Segment

This study examines surface deformation and fault activity along the Sındırgı segment using Ksn patterns together with fault-zone–scale structural field mapping. Ksn is used here as a proxy for relative changes in channel gradient that may reflect tectonic forcing; however, it is emphasized that high Ksn values alone are not uniquely diagnostic of active uplift and may also reflect lithologic contrasts, transient geomorphic adjustment, or drainage reorganization [68,69].

**Figure 11:**
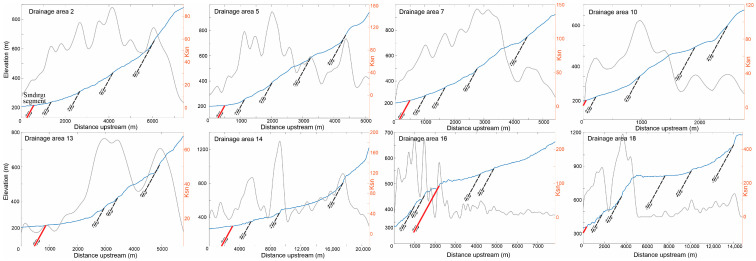
Longitudinal river profiles (blue solid lines) and normalized channel steepness (ksn) index values (grey solid lines) for streams within 19 drainage basins used in the morphometric analysis along the Sındırgı Segment. Locations of the drainage basins are shown in Figure 7. Active fault traces from Emre et al. [28] are shown by red solid lines, while black dashed lines indicate faults newly mapped in this study.

The mapped Ksn distribution shows pronounced spatial variability across the study area. Elevated Ksn values are concentrated along fault-bounded mountain fronts and upstream channel segments, particularly along the northern margin of the Sındırgı segment (Figure 11). These anomalies coincide spatially with mapped and newly identified fault traces, suggesting that deformation may be spatially heterogeneous and structurally influenced rather than regionally uniform. In contrast, lower Ksn values dominate inter-fault regions and southern drainage networks, where channel gradients are gentler and geomorphic adjustment appears more advanced.

Clusters of high Ksn values align with linear channel segments and knickpoints that are not fully explained by the previously mapped fault geometry. When considered together with field-based structural measurements, these patterns support the hypothesis that deformation is distributed across a segmented fault system, potentially involving secondary or partially buried structures. These inferred structures are depicted as dashed lineaments and are interpreted cautiously as candidate fault segments rather than confirmed active traces.

Field observations provide independent constraints that complement the surface deformation patterns identified in this study. Along-strike variations in measured slip-line pitch angles indicate changes in near-surface kinematics but do not uniquely resolve the depth extent or continuity of individual structures. These observations are broadly consistent with focal mechanism solutions from the 2025 earthquake sequence, suggesting the involvement of multiple fault elements while underscoring the limited coupling between surface expressions and coseismic rupture.

Taken together, the Ksn patterns, field mapping, and LiCSAR–InSAR observations support the testable hypothesis that deformation along the Sındırgı segment is distributed across a segmented fault system rather than localized on a single continuous structure. This hypothesis emphasizes the need for integrated analyses of geodetic strain rates and seismic moment release to better constrain how surface metrics relate to active deformation and seismic hazard.

## 6. Conclusions

This study examines the 2025 Sındırgı earthquake sequence and demonstrates that it is a clear example of fault reactivation redistributed by post-seismic stress. Based on the evidence presented here, the main findings, within their limitations, are summarized below.

1. Post-event field investigations following the 27 October 2025 earthquake documented surface cracks and shallow fractures, particularly in the Işıklar town. These observations are spatially consistent with deformation patterns inferred from InSAR and seismic data, providing independent confirmation of near-surface fault activity during the sequence.
2. The most significant result of this study is the documentation of distributed faulting along the Sındırgı segment. Rather than rupturing a single, continuous fault strand, the 2025 earthquake sequence involved multiple fault segments and subsidiary structures. This distributed fault network provides a plausible framework for explaining the spatially diffuse seismicity and the complexity of rupture propagation observed during the sequence.
3. InSAR observations resolved coseismic ground displacements of up to 7 cm, whereupon persistent post-seismic deformation was detected on the order of 10 mm/yr. These measurements demonstrate the capability of satellite geodesy to capture both transient and ongoing crustal deformation associated with moderate-magnitude earthquakes in structurally complex fault zones.
4. Aftershock distributions and deformation patterns indicate that seismic activity propagated onto faults that were previously unmapped or considered inactive. This observation suggests that earthquake sequences in the Sındırgı region may involve a broader deformation zone than implied by the primary mapped fault trace alone.

It is demonstrated that the current fault database is insufficient to explain the process behind the observed earthquake, as the stress-inversion seismogenic faults have not been delineated on the present maps. In this regard, integrating InSAR time-series analysis, stress inversion, and morphometric analysis has the potential to set the standard, whereby active-fault databases can be systematically updated. The improved method can help us compute multipurpose seismic hazard parameters in similar extensional tectonic provinces. While this study aims to contribute to a deeper understanding of the Sındırgı Segment and the 2025 earthquake sequence mechanism, one critical question still stands. Can post-seismic stress progression patterns be quantitatively estimated to determine which fault segments are likely to reactivate next, and if so, to what extent such reactivation is possible? Extending the methodology used in this paper to other geothermal areas might thenceforward assist in simulating deformation patterns in other tectonic and geothermal locations as GNNS technologies progress with additional recording stations, thereby strengthening the reliability of seismic hazard assessments.

## Figures and Tables

**Table 1:** Sentinel-1 LiCSAR interferograms between the epochs, including the moment of the 10 August Mw 6.1 earthquake and the 27 October 2025 Mw 6.1 Earthquake.

	Frame ID	Direction	Epochs Processed
10 August 2025 Mw 6.1 earthquake.	131A_05153_131313	Ascending	20250718–20250811
	036D_04976_131313	Descending	20250718–20250817
27 October 2025 Mw 6.1 Earthquake	131A_05153_131313	Ascending	20251016–20251028
	036D_04976_131313	Descending	20251016–20251028

**Table 2:** Parameters of stress inversion along the Sındırgı-Simav region.

Zone	Data	Used Data	σ1	σ2	σ3	R’	Tectonic Regime	SH_max_	St Dev	α	QRfmf
All	167	167	86/274	04/115	02/025	0.78	TT	115	12.3	15.6	B
Zone 1	40	40	02/123	87/241	03/033	1.08	TT	122	10.8	12.6	B
Zone 2	62	62	73/305	17/117	02/207	0.90	TT	117	11.2	12.6	B
Zone 3	65	65	86/158	02/287	03/017	0.64	PE	107	16.5	17.7	B

For each zone, the total number of events and the subset used in the stress tensor inversion are reported. The resulting reduced stress tensor is characterized by the plunge and strike (i.e., orientation) of the three principal stress axes (σ1, σ2, and σ3), along with the stress regime index (R’). Further parameters include the misfit angle (α) and the focal-mechanism stress inversion quality rank (QRfm) as per the classification scheme of the World Stress Map project. Stress regimes are denoted as TT for transtensional and PE for pure extensional conditions.

## Data Availability

All original data from this study are presented within the article. The datasets used are cited; direct retrieval from the original sources is recommended. Further data are available from the corresponding author upon reasonable request. Key seismic information, specifically focal mechanisms for earthquakes with a magnitude (M_W_) of 3.0 or larger, was sourced from the Turkish Disaster and Emergency Management Presidency (AFAD) (available at https://deprem.afad.gov.tr/event-focal-mechanism). InSAR data was provided by the LiCSAR system and made available through the COMET LiCSAR portal (https://comet.nerc.ac.uk/comet-lics-portal/, accessed on 28 November 2025).To convert wrapped phases into a continuous map of ground deformation, SNAPHU: Statistical-Cost, Network-Flow Algorithm for Phase Unwrapping (Version 2) was employed to perform phase unwrapping (available at https://web.stanford.edu/group/radar/softwareandlinks/sw/snaphu/, accessed on 28 November 2025). The great majority of the computational workload and figure generation rely primarily on MATLAB^®^ R2023b, licensed under an academic license from Karadeniz Technical University. Also, it is extended with two specialized toolboxes: TopoToolbox [67] Version 3 redeveloped within MATLAB^®^ 2023b environment and released in December 2025 (available at https://topotoolbox.wordpress.com/download/ and at https://github.com/TopoToolbox/topotoolbox3/releases), and MATLAB^®^-based Win-Tensor (Delvaux and Sperner [63]) software (latest version of 6.0.1 released in August 2025; available at https://damiendelvaux.be/Tensor/WinTensor/win-tensor_download.html). Spatial analysis and map production were conducted using both MATLAB^®^ and the ArcGIS^®^ platform, licensed through Karadeniz Technical University and Fırat University (available at https://www.esri.com/en-us/arcgis/about-arcgis/overview), and the open-source software called QGIS (version 3.34 LTR) (available at https://qgis.org/tr/site/forusers/download.html).
